# Supplementary material for: The Primary Cilium of Adipose Progenitors Is Necessary for Their Differentiation into Cancer-Associated Fibroblasts that Promote Migration of Breast Cancer Cells In Vitro
Source: Cells. 2020 Oct 8;9(10):2251. doi: 10.3390/cells9102251 (PMC7601294; doi:10.3390/cells9102251)
Supplement: Supplementary file 1 [file cells-09-02251-s001.pdf]

| | Forward | Reverse |
|---|---|---|
| TBP | CACGAACCACGGCACTGATT | TTTTCTTGCTGCCAGTCTGGAC |
| COL1A1 | ACCTGCGTGTACCCCACTCA | CCGCCATACTCGAACTGGAA |
| IFT88 | TGACATCTGCAAAACTCATTGCT | TCCACGCACCAATCATAACCT |
| TGF-b1 | GGAAACCCACAACGAAATCTATGA | GAGAGCAACACGGGTTCAGGTA |
| Leptin | AGGGAGACCGAGCGCTTTC | TGCATCTCCACACACCAAACC |
| PDGF-R | AGGTGGTTGACCTTCAATGG | TTTGATTTCTTCCAGCATTGTG |
| FAP | ATCTATGACCTTAGCAATGGAGAATTTGT | GTTTTGATAGACATATGCTAATTTACTCCCCAAC |
| FSP1 | TCCTCAGCGCTTCTTCTTTC | AACTTGTCACCCTCTTTGCC |
| HOXC8 | GTCTCCCAGCCTCATGTTTC | TCTGATACCGGCTGTAAGTTTGC |
| HOXC9 | CAGCAAGCACAAAGAGGAGA | CGACGGTCCCTGGTTAAATAC |

Supplementary Table: sequence of primer used for RT-QPCR

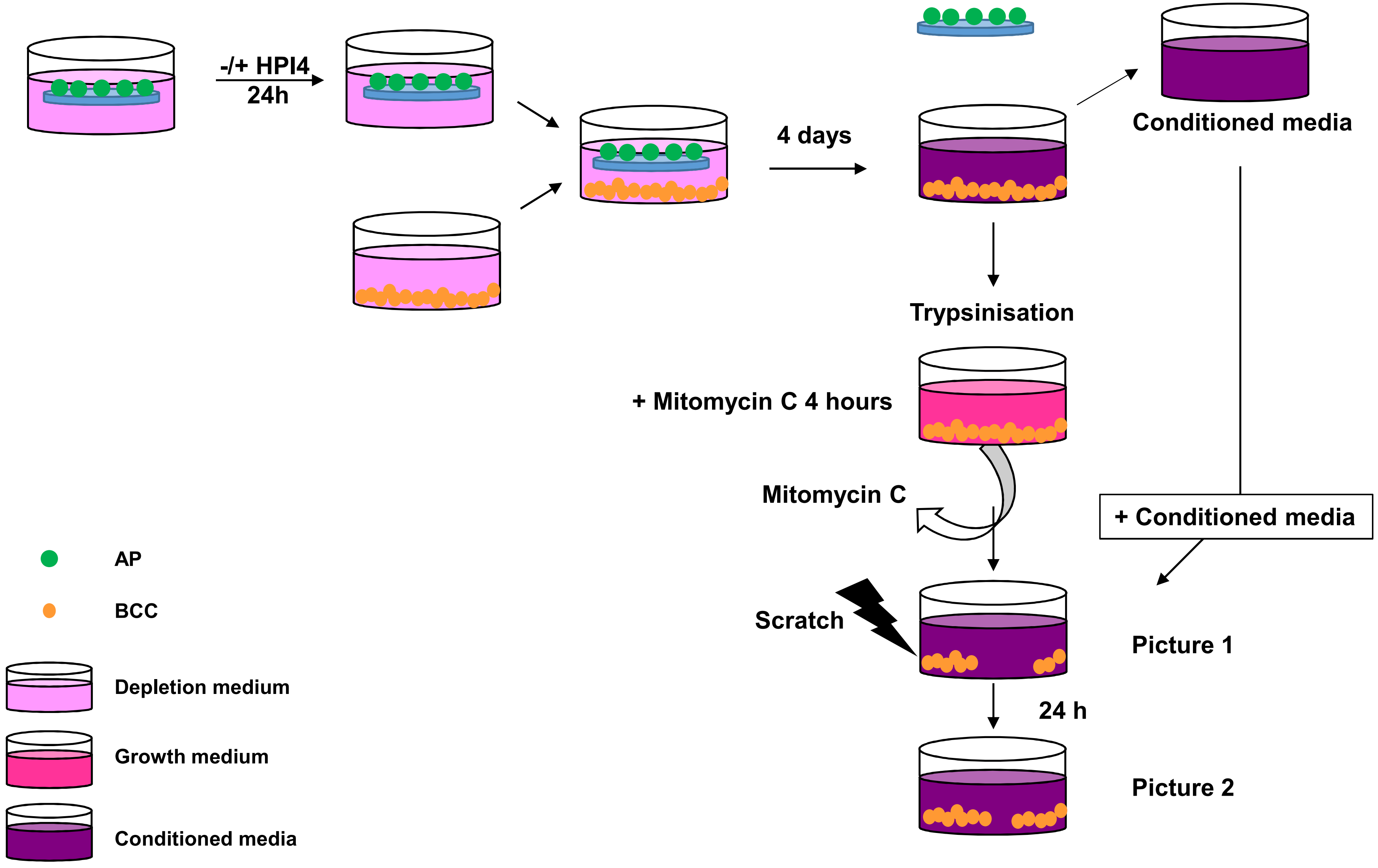


**Suplementary Figure 1:** Wound healing assay: APs were seeded on cover slips (18 mm diameter) and BCCs on 6 well dishes. After 24 h in depletion medium, APs were treated or not with HPI-4 for 24 h. HPI-4 was removed and cover slips containing APs were placed on BCCs. Controls, with cover slips without APs, were performed. After 4 days, cover slips were removed, conditioned media were recovered and BCCs were trypsinised and replated on two 24-wells in growth medium, in presence of mitomycin C (10µg/ml). After 4 h cells mitomycin C was removed and BCCs were incubated in their original conditioned medium and scratched with a pipet tip. Pictures were taken just after the scratch and 24 h later.

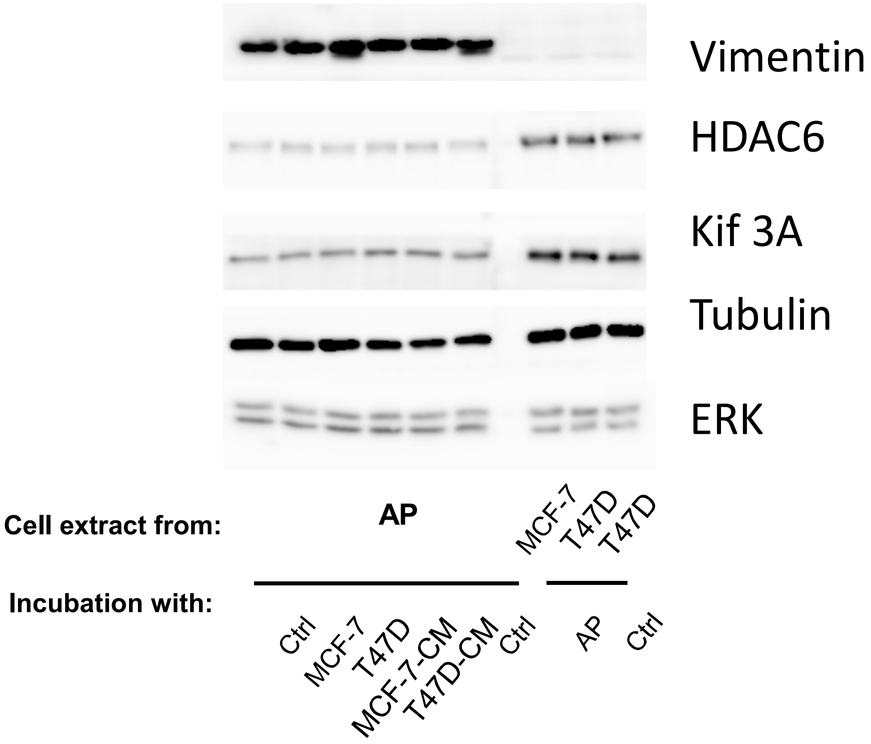


Suplementary Figure 2: human APs on cover slips were co-incubated in presence of control medium, MCF-7 or T47D cells or with conditioned medium (CM) from MCF-7 or T47D for 4 days. Proteins were extracted and analyzed by Western blot using the indicated antibodies.

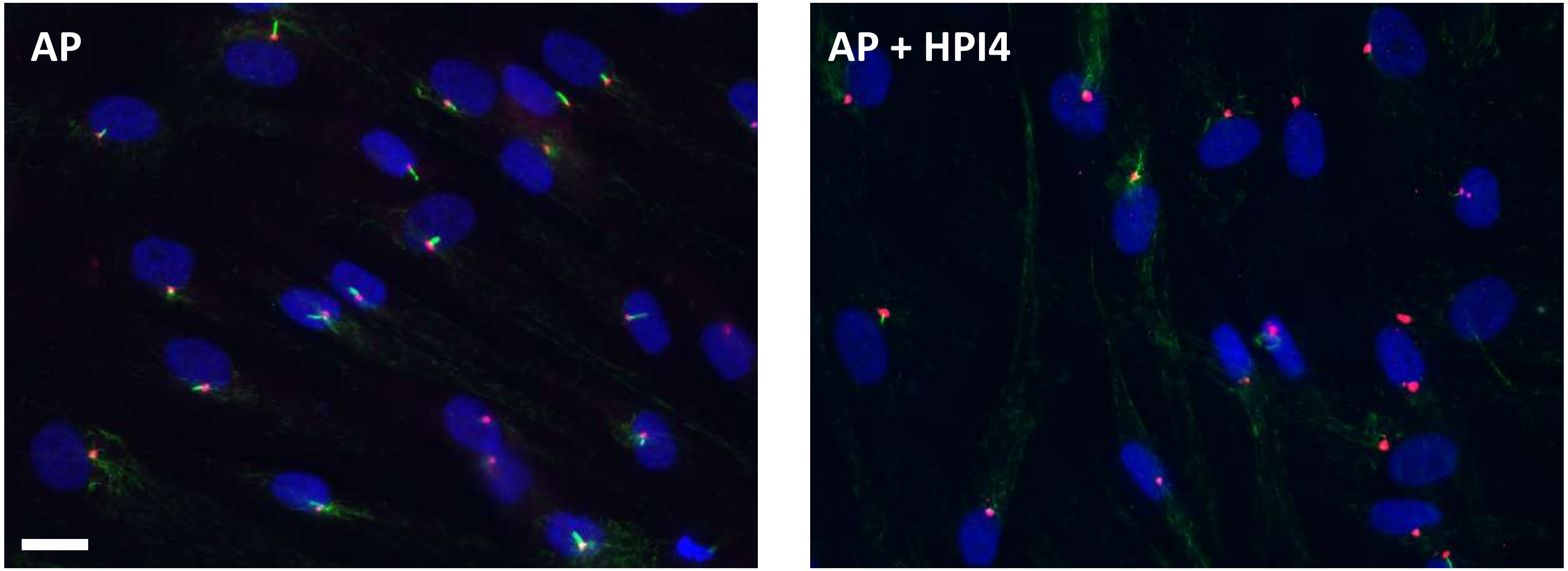


Supplementary Figure 3: Human APs on cover slips were treated or not with HPI-4 for 24 h. Cells were fixed and Acetylated tubulin (green) and Pericentrin (red) were revealed by immunocytochemistry, nuclei were stained with Hoechst 33258 (blue). The white bar represents 20 μM.
